# Concerns about ancient DNA data reported for Mengzi Ren, a Late Pleistocene individual from Southeast Asia

**DOI:** 10.1016/j.cub.2024.10.012

**Published:** 2025-03-24

**Authors:** Daniel Tabin, Nick Patterson, Matthew Mah, David Reich

**Affiliations:** 1 Department of Human Evolutionary Biology, Harvard University, Cambridge, Massachusetts 02138, USA; 2 Broad Institute of MIT and Harvard, Cambridge, Massachusetts 02142, USA; 3 Department of Genetics, Harvard Medical School, Boston, Massachusetts 02115, USA; 4 Howard Hughes Medical Institute, Boston, Massachusetts 02115, USA

## Abstract

Zhang et. al 2022 reported DNA sequences from an approximately 14-thousand-year-old skeleton excavated from Red Deer Cave: Mengzi Ren (MZR). MZR’s data are the first reported from pre-Holocene Southeast Asia, with genetic affinities dissimilar to all previously published ancient DNA data. We find extremely high error rates and an abnormal error distribution in the published sequences of MZR. Even ignoring these issues, we fail to replicate key population genetic findings from Zhang et al. These results raise concerns regarding the paper’s conclusions about population history and the usability of the published sequences.

## MZR has an extraordinarily high rate of atypical errors

**Figure 1:**
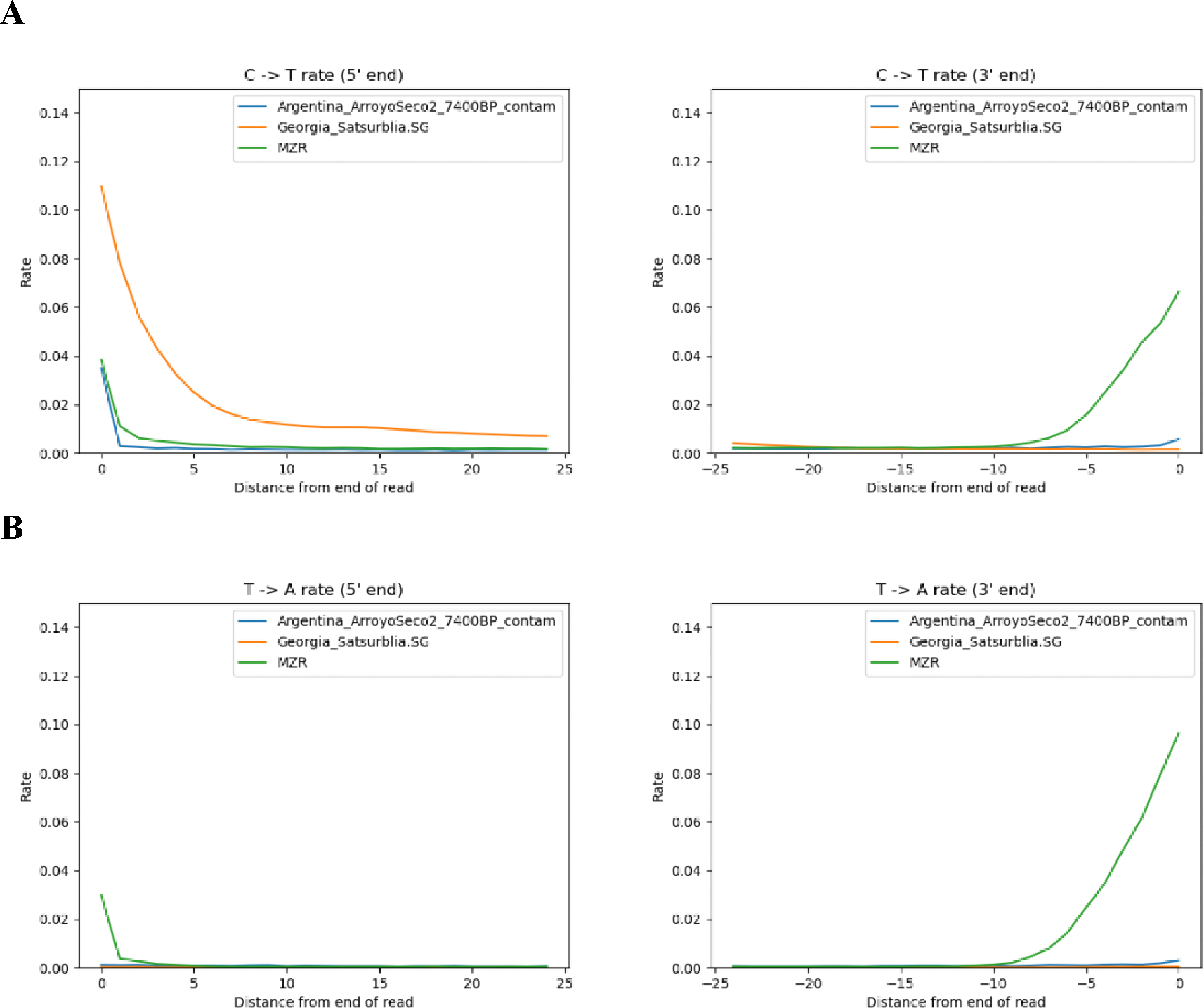
The MZR data show patterns of mismatch to the reference genome data unexpected for authentic ancient DNA data. (A) We examine C>T mismatches: positions that are cytosines in the human reference genome sequence but are misread as a thymine. At the 5’ end of sequences, we observe such characteristic signatures of ancient DNA at an elevated rate in MZR which superficially seems to be a signature of authenticity and indeed is also seen in two other ancient DNA datasets (a contaminated, low coverage ~7000-year-old ancient Argentinian, and a high-quality ~13000 year old ancient Georgian). However, at the 3’ end we observe a stronger mismatch rate than at the 5’ end, and at the 3’ end we also observe a slower falloff in the rate of mismatch as function of distance from the terminus than is the case at the 5’ end with substantially error rates in the final 8 base pairs; neither pattern is expected for known ancient DNA lab processes. (B) Elevated error rates, with stronger effects at the 3’ end extending to the final 8 bases, are also seen at other substitution classes like T>A not expected to show elevated rate of mismatches in authentic ancient DNA data. Data S1A, S1B, and S1C shows all substitution classes revealing additional instances of patterns unexpected for authentic ancient DNA data.

MZR’s genome^1^ has an extremely high rate of errors, and, particularly concerningly, these errors are not typical of those seen in typical ancient DNA. To illustrate the errors, Figure 1 and Data S1A shows the mismatch rate of both nuclear and mitochondrial DNA data to the reference sequence for MZR. There is an elevated rate of mismatch in the final nucleotides not only for cytosine deamination, which is expected in ancient DNA, but all substitution types (Data S1A, Data S1B, and Data S1C). Moreover, the error rate is often larger and the falloff as a function of distance from the end of sequence is slower on the 3’ than on the 5’ end of the sequence, which is not expected from known laboratory or bioinformatic processes. As an example of the impact, the 1^st^, 4^th^, 7^th^, and 8^th^ highest number of sequences supporting non-consensus alleles in MZR’s mitochondrial DNA are not even polymorphic in a diverse sample of 256 modern human mitochondrial genomes^2^ (Data S1D). A plausible explanation is that these sites have high error rates in the MZR data. There is no biochemical reason to think that an error process should be restricted to mitochondrial DNA; it plausibly applies to the whole genome as well.

## The published estimate of the contamination rate is unreliable

A powerful tool for verifying the authenticity of ancient DNA data is the mitochondrial genome, as it is not expected to be polymorphic in an uncontaminated individual, and it has a high copy number making it possible to determine a consensus sequence and thereby a rate of mismatch to the consensus. To analyze MZR’s mitochondrial DNA data, we first used *samtools*^3^ to remove fragments likely to be PCR duplicates, leaving an average coverage on mitochondrial DNA positions of 99. When we ran *ContamMix* on these data using standard settings, we estimate a 55–66% match rate to the consensus (95% confidence interval). This is in tension with the finding of the authors who inferred a much higher match rate to the mitochondrial consensus sequence of 94–100%. They obtained their estimate after restricting to a manually curated set of 14 positions which they chose because they recognized that the error rate in MZR’s sequences is extraordinarily high and sought to address this challenge by focusing on a subset of positions minimally affected by such errors. However, the error process in MZR’s data is so unusual that we are concerned it cannot be adequately addressed through filtering to a set of sites that are potentially less error prone. Moreover, trimming the sequences, either 8 bases per side, or 2 on the 5’ end and 17 on the 3’ end (Zhang et al.’s approach) does not address these issues as *ContamMix* estimates similar contamination in both cases.

The authors also report a direct estimate of autosomal contamination of 0.7% or less using the *AuthentiCT* software^4^, which appears to be in tension with our findings. However, the published MZR sequences do not have the characteristics required for *AuthentiCT*. *AuthentiCT* compares the damage profiles on the two ends of single-stranded ancient DNA libraries constructed without damage repair. Rates are expected to rise on both the 5’ and 3’ ends by similar amounts, with a correlation expected for contamination. However, the published MZR sequences show their damage predominantly on the 3’ end (Figure 1 and Data S1A). Whether this is due to a difference in biochemical processing compared to standard single-stranded libraries, or a bioinformatics issue, the conditions required by *AuthentiCT* are not met.

## The inference of MZR’s mitochondrial haplogroup is not reliable

The error rate in MZR’s mitochondrial data is so high it is not even clear what the consensus mitochondrial haplogroup is, let alone what the haplogroup of any potential contaminant would be. The authors infer that the haplogroup of MZR is basal M9 which they interpret as evidence that MZR carried a deeply divergent and previously unsampled Asian lineage. However, the consensus does not have all the expected derived alleles for M9 and contains derived alleles associated with different lineages as well as private alleles. This results in a low quality-score of 0.78 according to *haplogrep*^5^. Twenty other haplogroups, including the non-M haplogroups N and L3, have comparable quality scores (0.76–0.78) for the same data. We also aligned the sequences not to the inferred human ancestral mitochondrial sequence^6^, but instead to an M9 sequence constructed via a published M9a sequence^7^ from which we removed the mutations listed by Phylotree^8^ to reach basal M9. The new haplogroup inferred by *haplogrep* is even more basal: L3. The instability of haplogroup inference and evidence of abnormal and extremely high errors can also be seen in manual analysis. The diagnostic position for haplogroup M9 (G4491A) is not strongly supported, while the other allele correlated to this haplogroup (T16362C) is a recurrent mutation in the hypervariable region and thus cannot be seen as reliable support for M9. When trimming the reads following the strategy of Zhang et al with 2 bases on the 5’ and 17 bases on the 3’ end, these issues persist; for example, the most likely haplogroup according to *haplogrep* becomes R9 (a haplogroup outside of M), and the haplogroup quality-score remains low.

## Even if MZR’s data were authentic, they would not support a key conclusion

Even if we were to assume that the data from MZR accurately represent the ancestry of the population from which this individual came, MZR’s data do not in fact support one of its main conclusions that “there was an express northward expansion of AMHs starting in southern East Asia through the coastal line of China … eventually crossing the Bering Strait and reaching the Americas.” This finding was premised on a symmetry *f*_*4*_-statistic suggesting that Native Americans share alleles at an equal rate with Amur River Basin individuals from Northeast Asia from 19 kya^9^ and MZR, thus implying no more affinity of Native Americans to Late Pleistocene Northeast Asians than to Late Pleistocene Southeast Asians. However, when we recompute the statistics more powerfully (using our 8.65 million SNP set), Native Americans^10^ do in fact share significantly more ancestry with ~19kya Amur River Basin individuals than they do with MZR, as the symmetry statistic D(*MZR, AR19K; Ancient USA Anzick,Ancient Cameroon*) is Z = −3.6 standard errors below zero.

## Discussion

The ancestry of the people of the Red Deer Caves is important. While the MZR dataset has sufficiently high error rate and contamination that analysis is challenging, high quality data from MZR or other Red Deer Cave people has the potential to provide significant insights into the deep history of eastern non-Africans.

## Supplementary Material

Supplementary information

Supplementary Table **Data S1.** Tables highlighting MZR’s concerning patterns of mismatch to the reference genome sequence. (A) PMD tools full output for MZR, (B) for an ancient Georgian from Satsurblia, and (C) for an Argentinian contaminated sample. (D) Table of high-quality reads from MZR at mitochondrial sites with at least one high quality read not matching reference genome.
