# Supplementary material for: Concerns about ancient DNA data reported for Mengzi Ren, a Late Pleistocene individual from Southeast Asia
Source: Curr Biol. Author manuscript; Available in PMC 2025 Apr 29. (PMC12037273; doi:10.1016/j.cub.2024.10.012)
Supplement: Supplementary information [file NIHMS2073594-supplement-Supplementary_information.docx]

**Supplemental Information:**

**Concerns about ancient DNA data reported for Mengzi Ren, a Late Pleistocene individual from Southeast Asia**

Daniel Tabin,­ Nick Patterson, Matthew Mah, and David Reich

**Table S1: Z-scores for** $\boldsymbol{D}\left( \boldsymbol{Row, Column; USR}\boldsymbol{1, Mbuti} \right)$**.** MZR is in no way distinctive; many other ancient Northeast and Southeast Asians in the rows show positive Z-scores, reflecting shared ancestry with Native Americans relative to the outgroups in the columns.

**Table S2: Z-scores for** $\boldsymbol{D}\left( \boldsymbol{MZR, Northeast Asian;Native American;Cameroon SMA} \right)$**.** Native Americans show a higher affinity towards Northeast Asians than to MZR.


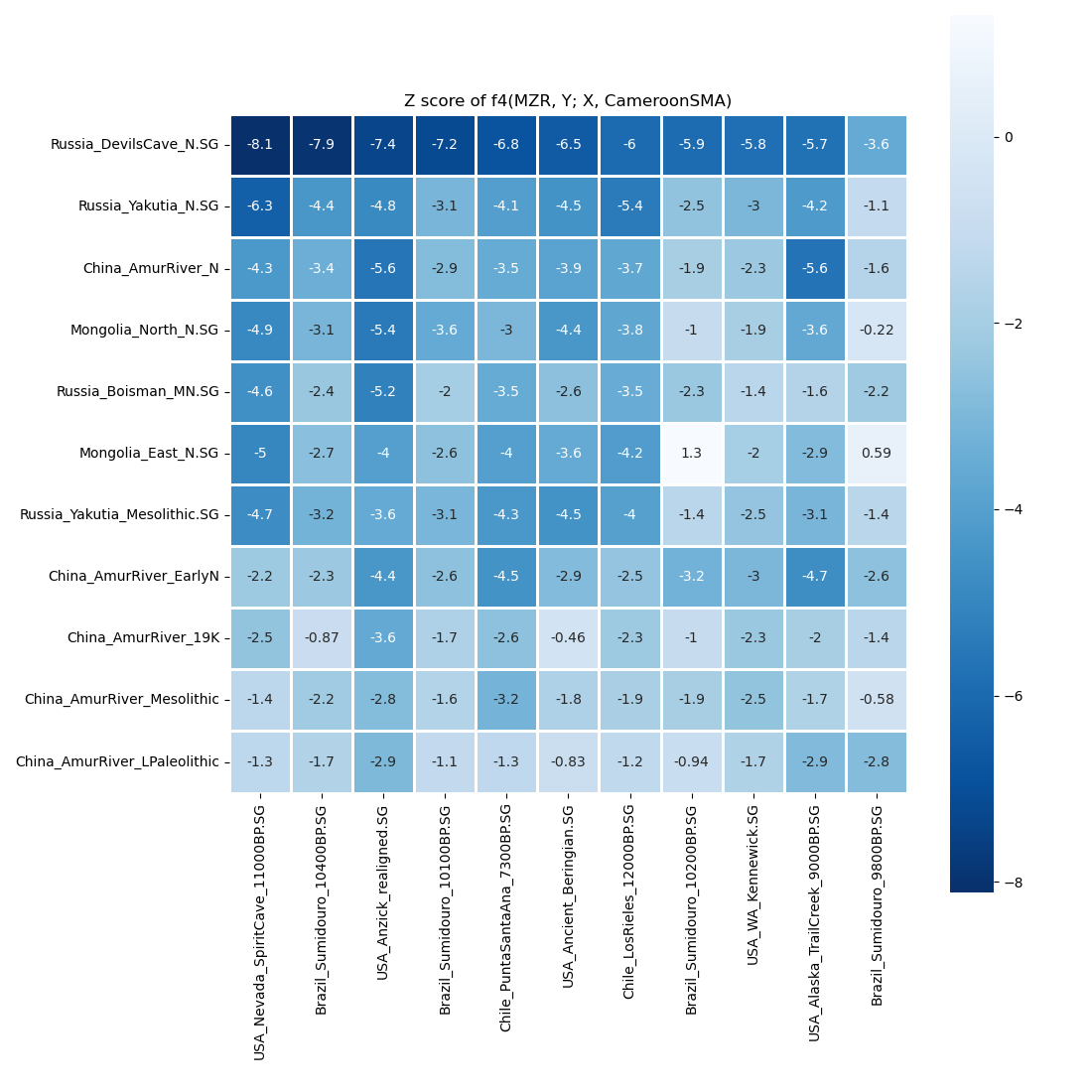


**Supplemental experimental procedures**

**Obtaining a larger SNP set**

To provide more power when analyzing MZR, we used the *cpoly* software[^1^](#_ENREF_1) to identify a set of ~86 million SNPs. We only use high coverage modern African samples to identify polymorphisms to avoid ascertainment bias. Additionally, we left Mbuti out as we were using them as an outgroup. The full list of African samples is given below:

T_Baka-1.DG T_Baka-2.DG T_Bakola-1.DG T_Bakola-2.DG S_BantuHerero-1.DG S_BantuHerero-2.DG HGDP01028.DG HGDP01035.DG HGDP00993.DG HGDP00994.DG HGDP01030.DG HGDP01033.DG HGDP01034.DG S_BantuTswana-1.DG S_BantuTswana-2.DG T_Bedzan-1.DG T_Bedzan-2.DG S_Biaka-1.DG S_Biaka-2.DG HGDP00454.DG HGDP00455.DG HGDP00457.DG HGDP00458.DG HGDP00459.DG HGDP00460.DG HGDP00461.DG HGDP00464.DG HGDP00466.DG HGDP00469.DG HGDP00470.DG HGDP00472.DG HGDP00473.DG HGDP00475.DG HGDP00985.DG HGDP00986.DG HGDP01086.DG HGDP01090.DG HGDP01094.DG T_Bulala-1.DG T_Bulala-2.DG A_Dinka-4.DG S_Dinka-1.DG B_Dinka-3.DG S_Dinka-2.DG S_Esan-2.DG S_Esan-1.DG HG02922.SG HG02923.SG HG02938.SG HG02941.SG HG02943.SG HG02944.SG HG02946.SG HG02947.SG HG02952.SG HG02953.SG HG02968.SG HG02970.SG HG02971.SG HG02973.SG HG02974.SG HG02976.SG HG02977.SG HG02979.SG HG02981.SG HG03099.SG HG03100.SG HG03103.SG HG03105.SG HG03108.SG HG03109.SG HG03111.SG HG03112.SG HG03114.SG HG03115.SG HG03117.SG HG03118.SG HG03120.SG HG03121.SG HG03123.SG HG03124.SG HG03126.SG HG03127.SG HG03129.SG HG03130.SG HG03132.SG HG03133.SG HG03135.SG HG03136.SG HG03139.SG HG03157.SG HG03159.SG HG03160.SG HG03162.SG HG03163.SG HG03166.SG HG03168.SG HG03169.SG HG03172.SG HG03175.SG HG03189.SG HG03190.SG HG03193.SG HG03195.SG HG03196.SG HG03198.SG HG03199.SG HG03202.SG HG03265.SG HG03267.SG HG03268.SG HG03270.SG HG03271.SG HG03279.SG HG03280.SG HG03291.SG HG03294.SG HG03295.SG HG03297.SG HG03298.SG HG03300.SG HG03301.SG HG03303.SG HG03304.SG HG03311.SG HG03313.SG HG03342.SG HG03343.SG HG03351.SG HG03352.SG HG03354.SG HG03363.SG HG03366.SG HG03367.SG HG03369.SG HG03370.SG HG03372.SG HG03499.SG HG03511.SG HG03514.SG HG03515.SG HG03517.SG HG03518.SG HG03520.SG HG03521.SG S_Igbo-1.DG S_Igbo-2.DG S_Ju_hoan_North-2.DG S_Ju_hoan_North-1.DG S_Ju_hoan_North-3.DG B_Ju_hoan_North-4.DG HGDP00987.DG HGDP00991.DG HGDP00992.DG HGDP01032.DG HGDP01036.DG T_Kaba-1.DG T_Kaba-2.DG S_Khomani_San-1.DG S_Khomani_San-2.DG S_Kongo-2.DG T_Laka-1.DG T_Laka-2.DG S_Lemande-1.DG S_Lemande-2.DG T_Mada-1.DG T_Mada-2.DG S_Mandenka-2.DG S_Mandenka-1.DG A_Mandenka-4.DG B_Mandenka-3.DG HGDP00904.DG HGDP00905.DG HGDP00906.DG HGDP00907.DG HGDP00908.DG HGDP00909.DG HGDP00910.DG HGDP00912.DG HGDP00913.DG HGDP00915.DG HGDP00917.DG HGDP01199.DG HGDP01200.DG HGDP01202.DG HGDP01283.DG HGDP01284.DG HGDP01286.DG S_Mende-1.DG S_Mende-2.DG HG03052.SG HG03054.SG HG03055.SG HG03057.SG HG03058.SG HG03060.SG HG03061.SG HG03063.SG HG03064.SG HG03066.SG HG03069.SG HG03072.SG HG03073.SG HG03074.SG HG03077.SG HG03078.SG HG03079.SG HG03081.SG HG03082.SG HG03084.SG HG03085.SG HG03086.SG HG03088.SG HG03091.SG HG03095.SG HG03096.SG HG03097.SG HG03209.SG HG03212.SG HG03224.SG HG03225.SG HG03376.SG HG03378.SG HG03380.SG HG03382.SG HG03385.SG HG03388.SG HG03391.SG HG03394.SG HG03397.SG HG03401.SG HG03410.SG HG03419.SG HG03428.SG HG03432.SG HG03433.SG HG03436.SG HG03437.SG HG03439.SG HG03442.SG HG03445.SG HG03446.SG HG03449.SG HG03451.SG HG03452.SG HG03455.SG HG03457.SG HG03458.SG HG03460.SG HG03461.SG HG03464.SG HG03469.SG HG03470.SG HG03472.SG HG03473.SG HG03476.SG HG03478.SG HG03479.SG HG03484.SG HG03485.SG HG03547.SG HG03548.SG HG03556.SG HG03557.SG HG03558.SG HG03559.SG HG03563.SG HG03565.SG HG03567.SG HG03571.SG HG03572.SG HG03575.SG HG03577.SG HG03578.SG HG03583.SG T_Ngumba-1.DG T_Ngumba-2.DG T_Tikar_South-1.DG T_Tikar_South-2.DG S_Yoruba-1.DG S_Yoruba-2.DG B_Yoruba-3.DG HGDP00920.DG HGDP00924.DG HGDP00925.DG HGDP00926.DG HGDP00928.DG HGDP00929.DG HGDP00930.DG HGDP00931.DG HGDP00932.DG HGDP00933.DG HGDP00934.DG HGDP00935.DG HGDP00936.DG HGDP00937.DG HGDP00938.DG HGDP00939.DG HGDP00940.DG HGDP00941.DG HGDP00942.DG HGDP00943.DG HGDP00944.DG NA18486.SG NA18488.SG NA18489.SG NA18498.SG NA18499.SG NA18501.SG NA18502.SG NA18504.SG NA18505.SG NA18507.SG NA18508.SG NA18510.SG NA18511.SG NA18516.SG NA18517.SG NA18519.SG NA18520.SG NA18522.SG NA18856.SG NA18858.SG NA18861.SG NA18864.SG NA18865.SG NA18867.SG NA18868.SG NA18870.SG NA18871.SG NA18873.SG NA18874.SG NA18876.SG NA18877.SG NA18878.SG NA18879.SG NA18881.SG NA18907.SG NA18908.SG NA18909.SG NA18910.SG NA18915.SG NA18916.SG NA18917.SG NA18923.SG NA18924.SG NA18933.SG NA18934.SG NA19093.SG NA19095.SG NA19096.SG NA19098.SG NA19099.SG NA19102.SG NA19107.SG NA19108.SG NA19113.SG NA19114.SG NA19116.SG NA19117.SG NA19118.SG NA19119.SG NA19121.SG NA19130.SG NA19131.SG NA19137.SG NA19138.SG NA19141.SG NA19143.SG NA19144.SG NA19146.SG NA19147.SG NA19149.SG NA19152.SG NA19153.SG NA19159.SG NA19160.SG NA19171.SG NA19175.SG NA19184.SG NA19185.SG NA19189.SG NA19190.SG NA19197.SG NA19198.SG NA19201.SG NA19204.SG NA19206.SG NA19207.SG NA19209.SG NA19210.SG NA19213.SG NA19214.SG NA19222.SG NA19223.SG NA19225.SG NA19235.SG NA19236.SG NA19238.SG NA19239.SG NA19247.SG NA19248.SG NA19256.SG NA19257.SG NA19093.DG NA19095.DG HG03139.DG HG03157.DG NA18867.DG NA18868.DG HG03129.DG HG03130.DG HG03363.DG HG03366.DG HG03499.DG HG03511.DG NA19107.DG NA19108.DG HG03517.DG HG03518.DG HG02974.DG HG02976.DG NA18499.DG NA18501.DG HG03291.DG HG03294.DG HG02968.DG HG02970.DG HG03313.DG HG03342.DG NA18486.DG NA18488.DG HG03066.DG HG03069.DG HG03472.DG HG03473.DG HG03428.DG HG03432.DG HG03304.DG HG03311.DG HG03458.DG HG03460.DG NA19113.DG NA19114.DG HG03120.DG HG03121.DG HG03270.DG HG03271.DG HG03301.DG HG03303.DG NA18881.DG NA18907.DG HG03123.DG HG03124.DG NA18489.DG NA18498.DG HG02952.DG HG02953.DG HG03195.DG HG03196.DG NA19096.DG NA19098.DG NA19099.DG NA19102.DG HG03451.DG HG03452.DG HG03091.DG HG03095.DG NA19257.DG HG03086.DG HG03088.DG HG03367.DG HG03369.DG HG03352.DG HG03354.DG NA18508.DG NA18510.DG HG03081.DG HG03082.DG HG03572.DG HG03575.DG HG03469.DG HG03470.DG HG03169.DG HG03172.DG HG03111.DG HG03112.DG NA19222.DG NA19223.DG NA19130.DG NA19131.DG HG03108.DG HG03109.DG HG03132.DG HG03133.DG HG03057.DG HG03058.DG HG03114.DG HG03115.DG HG03190.DG HG03193.DG HG03437.DG HG03439.DG NA18873.DG NA18874.DG NA18856.DG HG03376.DG HG03378.DG NA18876.DG NA18877.DG HG03298.DG HG03300.DG HG03442.DG HG03445.DG NA19225.DG NA19235.DG NA19152.DG NA19153.DG NA19206.DG NA19207.DG HG03166.DG HG03168.DG NA19248.DG NA19256.DG HG03162.DG HG03163.DG NA19171.DG HG03202.DG HG03209.DG NA18858.DG NA18861.DG HG03455.DG HG03457.DG NA18915.DG NA18916.DG HG03343.DG HG03351.DG HG03159.DG HG03160.DG NA18505.DG NA18507.DG NA19236.DG NA19238.DG HG03052.DG NA18511.DG NA18516.DG NA18878.DG NA18879.DG HG03485.DG HG03175.DG HG03189.DG HG03265.DG HG03391.DG HG03394.DG NA19137.DG NA19138.DG HG03370.DG HG03372.DG HG02946.DG HG02947.DG HG03084.DG HG03085.DG HG03267.DG HG03268.DG HG03295.DG HG03297.DG HG03410.DG HG03419.DG NA19190.DG NA19197.DG NA18924.DG NA18933.DG NA19209.DG NA19210.DG HG03126.DG HG03127.DG NA19141.DG NA19143.DG HG03563.DG HG03565.DG HG03103.DG HG03105.DG NA18502.DG NA18504.DG HG02971.DG HG02973.DG NA18917.DG NA18923.DG HG03577.DG HG03578.DG HG03397.DG HG03401.DG NA18517.DG NA18519.DG HG03279.DG HG03280.DG HG02922.DG HG02923.DG HG02981.DG NA18908.DG NA18909.DG HG03520.DG HG03521.DG HG03446.DG HG03449.DG NA19144.DG NA19146.DG HG03567.DG HG03571.DG HG03479.DG HG03484.DG NA19116.DG NA19117.DG HG03096.DG HG03097.DG NA19147.DG NA19149.DG NA18910.DG HG03063.DG HG03064.DG HG03476.DG HG03478.DG HG03433.DG HG03436.DG NA19159.DG NA19160.DG HG03514.DG HG03515.DG HG03583.DG NA19239.DG NA19247.DG HG03547.DG HG03548.DG HG03198.DG HG03199.DG NA19213.DG NA19214.DG HG03099.DG HG03100.DG HG03072.DG HG03073.DG NA19121.DG NA19198.DG NA19118.DG NA19119.DG HG02977.DG HG02979.DG HG02938.DG HG02941.DG HG03556.DG HG03557.DG NA19175.DG NA19184.DG HG03212.DG HG03224.DG HG03054.DG HG03055.DG HG03380.DG HG03382.DG HG03060.DG HG03061.DG HG03385.DG HG03388.DG HG02943.DG HG02944.DG HG03461.DG HG03464.DG NA18520.DG NA18522.DG NA19201.DG NA19204.DG NA18864.DG NA18865.DG HG03225.DG HG03558.DG HG03559.DG NA19185.DG NA19189.DG HG03074.DG HG03077.DG HG03117.DG HG03118.DG HG03078.DG HG03079.DG HG03135.DG HG03136.DG NA18934.DG NA18870.DG NA18871.DG

We merged these SNPs with a set of SNPs around the non-Archaic probes from Fu et al. 2015[^2^](#_ENREF_2) as well as the off target SNPs as described in Rohland 2022[^3^](#_ENREF_3). This gave 86 million SNPs.

**Obtaining ancient DNA data overlapping the 86 million SNPs**

Once we had produced a working SNP file, we obtained the genotype for every sample at each locus by randomly sampling a single genotype from all of the reads overlapping that position*.* This produced a snp, ind, and geno file for each individual in EIGENSTRAT format[^4^](#_ENREF_4). We generated “pseudohaploid” call based on the randomly sampled alleles for each individual at each position, assigning them to be “0” “2” or “9” representing homozygous ancestral, homozygous derived, and missing respectively.

**Merging Geno files**

We merged individuals into a single set of SNP ind and geno files by concatenating the geno files using *paste*, and the ind files with *cat* in linux.

**Removing extra SNPS**

While every SNP in the 86 million SNP set was polymorphic in the African samples, not every SNP was polymorphic in the data we analyzed for this study. We used *awk* in linux to remove every line of our SNP and geno files that lacked both a “0” and a “2” representing at least one individual with the ancestral and derived SNP. This does not change the Z-scores, as the number of ABBAs and BABAs that go into f-statistics are inherently unchanged by this process (which for any set of populations only removes AAAAs and BBBBs). This left 8,650,283 SNPs on which we performed analyses.

**Supplemental results and discussion**

**Evidence against the claim that Native Americans are equally related to MZR and Amur River Hunter Gatherers from 19 kya**

Zhang et al.[^5^](#_ENREF_5) provide two main pieces of evidence for MZR having more affinity to Native Americans that later Southeast Asians, which is the basis for their proposal of “an express northward expansion of AMHs starting in southern East Asia through the coastal line of China … eventually crossing the Bering Strait and reaching the Americas”[^5^](#_ENREF_5).

First, Zhang et al. cite statistics of the form $D(Row, Column; USR1, Mbuti)$, which measure whether Native Americans, represented by an Ancient Beringian from Alaska USR1[^6^](#_ENREF_6), share more alleles with a “Row” population or a “Column” population corresponding to mostly Upper Paleolithic Eurasians (Table S1), using sub-Saharan Africans (Mbuti)[^1^](#_ENREF_1)^,^[^7^](#_ENREF_7) as an outgroup assumed to be equidistant to all non-Africans. Zhang et al. observe that when Row = MZR, the quantity is significantly greater than $0$, and we verified this for a diverse range of early Eurasian hunter-gatherers[^8-13^](#_ENREF_8) (Table S1). However, this genetic affinity to Native Americans is not unique to MZR: similar patterns manifest for the majority of Holocene and Late Pleistocene East Asians[^1^](#_ENREF_1)^,^[^7^](#_ENREF_7)^,^[^10^](#_ENREF_10)^,^[^11^](#_ENREF_11)^,^[^13^](#_ENREF_13)^,^[^14^](#_ENREF_14), for whom all Column Populations are outgroups in a phylogenetic sense (Table S1). Thus, there is nothing distinctive about the values this D-statistic takes that would suggest a Late Pleistocene expansion starting as far south as southern East Asia, and ultimately contributing to Native Americans. The values manifested by this D-statistic in Table S1 can be explained by a plausible scenario in which the East Asian-associated ancestry in Native Americans comes from Northeast Asia[^15^](#_ENREF_15), whose populations since the Last Glacial Maximum have shared genetic drift with the great majority of Holocene and Late Pleistocene East Asians compared to Column Populations.

The second line of evidence cited by Zhang et al. in support of a Southeast Asian migration to the Americas comes from their inference that Native Americans have no more affinity to an individual from the Amur River Basin in northeast Asia from ~19 kya than to MZR. They write: “MZR [and] Amur-19.0K are cladal with respect to First Americans, suggesting the East Asian contribution to Native Americans likely originated prior to the south-versus-north East Asian divergence” In other words, Zhang et al. suggest that the 19 kya Amur River Basin genome—dated to five thousand years before MZR, buried thousands of kilometers to the north, and hypothesized to be related to the East Asian lineage from whom Native Americans harbor ancestry[^11^](#_ENREF_11)—may not harbor lineages that are closer to Native Americans in a phylogenetic sense than to MZR. If this were true, then there would indeed be no genetic evidence for a northern rather than a southern origin for the East Asian-associated lineages present in Native Americans.

To support their argument, Zhang et al. cite symmetry statistics of the form D$\left( MZR, AR19K; Native American, African \right),$ measuring which of the first two listed populations shares more alleles with Native Americans. Zhang et al. find this statistic is non-significantly different from zero (Z = -0.656), consistent with being a clade. However, an alternative reason for Z-scores of low magnitude is limited data from MZR, which reduces statistical power, and reliance on only a single Native American sample, USR1. We carried out all our analyses on a larger set of 8.65 million SNPs that we ascertained to increase power (Supplementary File 2), represented Native Americans with multiple samples[^6^](#_ENREF_6)^,^[^16^](#_ENREF_16)^,^[^17^](#_ENREF_17), and represented Africans with high-quality ancient data from Cameroon[^18^](#_ENREF_18) to avoid biases derived from mixing ancient and modern data. We additionally tested ancient Northeast Asians[^6^](#_ENREF_6)^,^[^11^](#_ENREF_11)^,^[^19^](#_ENREF_19) other than AR19K. Table S2 shows that Native Americans are significantly more closely related to AR19K than to MZR; for example, D$(MZR, AR19K; USA\_Anzick,Cameroon)$ is Z = -3.6 standard errors below zero, reflecting excess allele sharing between AR19K and Native Americans, contradicting the conjecture of Zhang et al. This finding replicates when the MZR reads are aggressively trimmed in order to reduce the high amount of noise mentioned in the main manuscript (we tried trimming 8 base pairs on either side, as well as 2 base pairs on the 5’ and 17 base pairs on the 3’ side as Zhang et al. did, and obtained qualitatively similar results).

**Conclusions**

Our analyses show that, contrary to claims by Zhang et al, at least some of the East Asian ancestry in Native Americans likely split off from Northeast Asians after the split with the ancestors of Southeast Asians including MZR. While it remains possible that Native Americans harbor some Southeast Asian ancestry, analyses of MZR data provide no evidence for this.

**Supplemental References**

S1. Mallick, S., Li, H., Lipson, M., Mathieson, I., Gymrek, M., Racimo, F., Zhao, M., Chennagiri, N., Nordenfelt, S., Tandon, A., et al. (2016). The Simons Genome Diversity Project: 300 genomes from 142 diverse populations. Nature *538*, 201-206. 10.1038/nature18964.

S2. Fu, Q., Hajdinjak, M., Moldovan, O.T., Constantin, S., Mallick, S., Skoglund, P., Patterson, N., Rohland, N., Lazaridis, I., Nickel, B., et al. (2015). An early modern human from Romania with a recent Neanderthal ancestor. Nature *524*, 216-219. 10.1038/nature14558.

S3. Rohland, N., Mallick, S., Mah, M., Maier, R., Patterson, N., and Reich, D. (2022). Three assays for in-solution enrichment of ancient human DNA at more than a million SNPs. Genome Res *32*, 2068-2078. 10.1101/gr.276728.122.

S4. Price, A.L., Patterson, N.J., Plenge, R.M., Weinblatt, M.E., Shadick, N.A., and Reich, D. (2006). Principal components analysis corrects for stratification in genome-wide association studies. Nat Genet *38*, 904-909. 10.1038/ng1847.

S5. Zhang, X., Ji, X., Li, C., Yang, T., Huang, J., Zhao, Y., Wu, Y., Ma, S., Pang, Y., Huang, Y., et al. (2022). A Late Pleistocene human genome from Southwest China. Curr Biol *32*, 3095-3109 e3095. 10.1016/j.cub.2022.06.016.

S6. Moreno-Mayar, J.V., Potter, B.A., Vinner, L., Steinrucken, M., Rasmussen, S., Terhorst, J., Kamm, J.A., Albrechtsen, A., Malaspinas, A.S., Sikora, M., et al. (2018). Terminal Pleistocene Alaskan genome reveals first founding population of Native Americans. Nature *553*, 203-207. 10.1038/nature25173.

S7. Prufer, K., Racimo, F., Patterson, N., Jay, F., Sankararaman, S., Sawyer, S., Heinze, A., Renaud, G., Sudmant, P.H., de Filippo, C., et al. (2014). The complete genome sequence of a Neanderthal from the Altai Mountains. Nature *505*, 43-49. 10.1038/nature12886.

S8. Fu, Q., Li, H., Moorjani, P., Jay, F., Slepchenko, S.M., Bondarev, A.A., Johnson, P.L., Aximu-Petri, A., Prufer, K., de Filippo, C., et al. (2014). Genome sequence of a 45,000-year-old modern human from western Siberia. Nature *514*, 445-449. 10.1038/nature13810.

S9. Sikora, M., Seguin-Orlando, A., Sousa, V.C., Albrechtsen, A., Korneliussen, T., Ko, A., Rasmussen, S., Dupanloup, I., Nigst, P.R., Bosch, M.D., et al. (2017). Ancient genomes show social and reproductive behavior of early Upper Paleolithic foragers. Science *358*, 659-662. 10.1126/science.aao1807.

S10. Sikora, M., Pitulko, V.V., Sousa, V.C., Allentoft, M.E., Vinner, L., Rasmussen, S., Margaryan, A., de Barros Damgaard, P., de la Fuente, C., Renaud, G., et al. (2019). The population history of northeastern Siberia since the Pleistocene. Nature *570*, 182-188. 10.1038/s41586-019-1279-z.

S11. Mao, X., Zhang, H., Qiao, S., Liu, Y., Chang, F., Xie, P., Zhang, M., Wang, T., Li, M., Cao, P., et al. (2021). The deep population history of northern East Asia from the Late Pleistocene to the Holocene. Cell *184*, 3256-3266 e3213. 10.1016/j.cell.2021.04.040.

S12. Fu, Q., Meyer, M., Gao, X., Stenzel, U., Burbano, H.A., Kelso, J., and Paabo, S. (2013). DNA analysis of an early modern human from Tianyuan Cave, China. Proc Natl Acad Sci U S A *110*, 2223-2227. 10.1073/pnas.1221359110.

S13. McColl, H., Racimo, F., Vinner, L., Demeter, F., Gakuhari, T., Moreno-Mayar, J.V., van Driem, G., Gram Wilken, U., Seguin-Orlando, A., de la Fuente Castro, C., et al. (2018). The prehistoric peopling of Southeast Asia. Science *361*, 88-92. 10.1126/science.aat3628.

S14. Wang, T., Wang, W., Xie, G., Li, Z., Fan, X., Yang, Q., Wu, X., Cao, P., Liu, Y., Yang, R., et al. (2021). Human population history at the crossroads of East and Southeast Asia since 11,000 years ago. Cell *184*, 3829-3841 e3821. 10.1016/j.cell.2021.05.018.

S15. Yu, H., Spyrou, M.A., Karapetian, M., Shnaider, S., Radzeviciute, R., Nagele, K., Neumann, G.U., Penske, S., Zech, J., Lucas, M., et al. (2020). Paleolithic to Bronze Age Siberians Reveal Connections with First Americans and across Eurasia. Cell *181*, 1232-1245 e1220. 10.1016/j.cell.2020.04.037.

S16. Rasmussen, M., Anzick, S.L., Waters, M.R., Skoglund, P., DeGiorgio, M., Stafford, T.W., Jr., Rasmussen, S., Moltke, I., Albrechtsen, A., Doyle, S.M., et al. (2014). The genome of a Late Pleistocene human from a Clovis burial site in western Montana. Nature *506*, 225-229. 10.1038/nature13025.

S17. Rasmussen, M., Sikora, M., Albrechtsen, A., Korneliussen, T.S., Moreno-Mayar, J.V., Poznik, G.D., Zollikofer, C.P.E., de Leon, M.P., Allentoft, M.E., Moltke, I., et al. (2015). The ancestry and affiliations of Kennewick Man. Nature *523*, 455-458. 10.1038/nature14625.

S18. Lipson, M., Sawchuk, E.A., Thompson, J.C., Oppenheimer, J., Tryon, C.A., Ranhorn, K.L., de Luna, K.M., Sirak, K.A., Olalde, I., Ambrose, S.H., et al. (2022). Ancient DNA and deep population structure in sub-Saharan African foragers. Nature *603*, 290-296. 10.1038/s41586-022-04430-9.

S19. Zeng, T.C., Vyazov, L.A., Kim, A., Flegontov, P., Sirak, K., Maier, R., Lazaridis, I., Akbari, A., Frachetti, M., Tishkin, A.A., et al. (2023). 10.1101/2023.10.01.560332.
